# Predictors of Recurrent *Clostridioides difficile* Infection

**DOI:** 10.3390/diagnostics16070969

**Published:** 2026-03-24

**Authors:** Samuel Bogdan Todor, Adrian Boicean, Minodora Teodoru, Paula Anderco, Oana Stoia, Mirela Livia Popa, Cristian Ichim

**Affiliations:** Faculty of Medicine, Lucian Blaga University of Sibiu, 550169 Sibiu, Romania; samuelbogdant@gmail.com (S.B.T.); adrian.boicean@ulbsibiu.ro (A.B.); oana.stoia@ulbsibiu.ro (O.S.); liviamirelapopa@yahoo.com (M.L.P.); cristian.ichim@ulbsibiu.ro (C.I.)

**Keywords:** *Clostridioides difficile* infection, antibiotic exposure, recurrence, risk factors

## Abstract

**Background:** Recurrence remains a major challenge in the management of *Clostridioides difficile* infection (CDI), with reported rates of 20–30% after an index episode. Identification of factors associated with recurrence is essential for improved risk stratification. **Methods:** This retrospective cohort study included 100 adult patients diagnosed with CDI. Factors associated with recurrent CDI were evaluated using univariable analyses, receiver operating characteristic analysis and backward stepwise logistic regression. **Results:** Eighteen patients (18%) developed recurrent CDI. Baseline demographic characteristics, comorbidity burden, clinical presentation and admission laboratory parameters were not significantly associated with recurrence. Previous hospitalization within the preceding 12 months, longer duration of antibiotic therapy and poor or partial response to initial treatment were independently associated with recurrence. Duration of antibiotic treatment showed the strongest discriminatory performance (AUC 0.712). Predictive models combining treatment response, antibiotic duration and prior hospitalization demonstrated incremental improvement in discrimination, achieving an AUC of 0.775. Associations with specific antibiotic classes did not persist after adjustment for healthcare exposure and treatment duration. **Conclusions:** Recurrent CDI was associated primarily with healthcare exposure and post-diagnosis treatment characteristics rather than baseline clinical or laboratory features. These findings support the relevance of integrating antibiotic burden and early treatment response into recurrence risk assessment. However, the relatively small number of recurrent cases warrants cautious interpretation of these findings.

## 1. Introduction

*Clostridioides difficile* is a Gram-positive, spore-forming, obligate anaerobic bacterium within the Firmicutes phylum [1]. Initially isolated in 1935 from the intestinal microbiota of healthy neonates, the organism was long regarded as non-pathogenic and was first designated Bacillus difficilis due to its demanding growth conditions before being reclassified under its current nomenclature [2,3]. The pathogenic relevance of the bacteria was not fully established until the late 1970s [4,5]. Primary infection is strongly linked to prior antibiotic exposure, which disrupts the normal anaerobic gut flora and creates a permissive environment for *Clostridioides difficile* colonization and expansion [6,7,8]. The pathogenicity of difficile is primarily mediated by toxins A and B, which inactivate Rho GTPases, disrupt epithelial signaling, and impair intestinal barrier integrity [9,10,11]. These toxins also induce a marked inflammatory response through neutrophil and mast cell recruitment and cytokine release [12]. In severe cases, systemic dissemination may occur, leading to toxemia and extraintestinal complications, although documented human cases remain rare [11,13,14,15]. Limited sensitivity of current assays for circulating toxins likely contributes to underrecognition of CDI-associated toxemia in clinical practice [13,14,15,16,17,18].

*Clostridioides difficile* infection (CDI) is one of the most prevalent healthcare-associated infections, with a notable rise in incidence and severity over the past decade [19]. With an estimated incidence of 110.2 cases per 100,000 individuals, CDI remains a major cause of infectious-diarrhea-related morbidity and mortality in the United States [20]. Although traditionally linked to hospital settings and classified among the top public health threats by the Centers for Disease Control and Prevention, recent population-based data indicate that up to 41% of cases are acquired in the community [21,22]. While community-onset CDI generally follows a milder course, its epidemiological relevance remains significant, highlighting the importance of early risk identification beyond hospital environments [23].

The disease develops following disruption of the normal colonic microbiota, which permits uncontrolled expansion of toxin-producing, Gram-positive, spore-forming anaerobic bacilli [24]. CDI risk is strongly influenced by antibiotic exposure, advanced age, hospitalization and immunosuppression. Older patients, particularly those with multiple comorbidities, face a substantially higher risk of complications and mortality [25]. Antibiotic use during hospitalization remains one of the most important and well-established modifiable risk factors for CDI development [26,27].

Antibiotic exposure, the principal predisposing factor for CDI, reduces protective anaerobic bacterial populations, thereby eliminating colonization resistance and facilitating *C. difficile* proliferation [28]. Disease severity is further influenced by impaired host immune responses, leading to inadequate bacterial clearance and exaggerated toxin-mediated inflammation [24,29,30,31]. Clinically, CDI typically presents with fever, leukocytosis, abdominal pain and watery diarrhea, while severe forms may progress to dehydration, electrolyte disturbances, acute kidney injury, pseudomembranous colitis or fulminant disease characterized by ileus, toxic megacolon or shock, necessitating urgent intensive treatment [32,33,34].

Once CDI is established, disease severity and recurrence history are key determinants of treatment strategy. In addition to standard antimicrobial therapy, bezlotoxumab may reduce the risk of recurrent CDI in high-risk patients, including older adults, immunocompromised individuals, and those with recent or severe disease [35,36,37]. Recurrence rates increase markedly after multiple episodes, partly due to further microbiota disruption caused by repeated antibiotic exposure [38]. Consequently, microbiota restoration therapies are increasingly considered. While fecal microbiota transplantation remains guideline-recommended, newly approved live biotherapeutic products offer standardized alternatives, with real-world data needed to clarify their role in clinical practice [39,40]. Although several risk factors for recurrent CDI have been identified, including advanced age, antibiotic exposure, and healthcare-associated infection, findings remain inconsistent across studies, particularly regarding the relative contribution of baseline clinical characteristics versus post-diagnosis factors such as treatment response and antibiotic burden. Moreover, most predictive models rely on heterogeneous variables and show variable performance across clinical settings.

The aim of this study was to identify factors associated with recurrent CDI, with particular focus on healthcare exposure and antibiotic treatment characteristics.

We hypothesized that post-diagnosis factors, particularly antibiotic treatment duration and early treatment response, may have a stronger predictive value for recurrence than baseline clinical or laboratory parameters.

## 2. Materials and Methods

We conducted a retrospective, single-center observational study at the County Clinical Emergency Hospital of Sibiu. The study population consisted of adult patients hospitalized with a diagnosis of CDI. A total of 100 consecutive patients admitted during the 6-year study (1 January 2020–1 January 2026) period were included in the analysis in order to reduce selection bias and maximize the use of the available dataset.

Eligibility criteria included age ≥18 years and a confirmed diagnosis of CDI, defined by the presence of compatible clinical symptoms and a positive stool test for *C. difficile*. (CDI diagnosis was established using an enzyme immunoassay (EIA) for toxin A/B detection, performed in the hospital laboratory; however, specific information regarding the assay manufacturer was not available due to the retrospective nature of the study.) Patients with incomplete clinical or laboratory data were excluded from the study. Only index CDI episodes were considered for inclusion. Given the retrospective nature of the study, the requirement for informed consent was waived.

The primary outcome of interest was recurrent CDI, defined as the reappearance of compatible clinical symptoms with laboratory confirmation of *C. difficile* following initial resolution of the index episode.

Clinical and laboratory data were collected retrospectively from the hospital’s electronic medical records. Extracted variables included demographic characteristics, healthcare exposure, and medication exposure prior to CDI. Antibiotic exposure was recorded both as overall exposure and by individual antibiotic classes, along with the number and duration of antibiotic treatments. Antibiotics were categorized by pharmacological class (e.g., penicillins, carbapenems, macrolides, fluoroquinolones), while cephalosporins were included within the broader beta-lactam category but were not analyzed separately due to limited frequency. Use of proton pump inhibitors, corticosteroids, chemotherapy, biological therapies, probiotics and nutritional support was also documented. Clinical predictors included demographic characteristics, comorbidities, prior healthcare exposure, medication use and clinical presentation variables recorded before or during the index CDI episode.

Recurrence was defined as a new episode of CDI occurring within 8 weeks after completion of treatment for the initial episode, in accordance with current clinical guidelines. Antibiotics were categorized by pharmacological class (penicillins, carbapenems, macrolides, fluoroquinolones, etc.), and agents with low frequency were not analyzed separately. Recurrence episodes were identified based on readmissions or documented follow-up visits within the same hospital electronic medical records. Episodes managed in other healthcare settings may not have been captured, representing a potential source of underestimation.

Comorbidity burden was assessed using the Charlson Comorbidity Index, and individual comorbid conditions were recorded. Clinical presentation, treatment regimens, treatment response (classified as good or poor/partial), complications, and length of hospitalization were analyzed. A good clinical response was defined as complete resolution of diarrhea (≤3 unformed stools per 24 h) and improvement of associated symptoms by the end of therapy, without the need for treatment modification. Poor or partial clinical response was defined as persistence or incomplete resolution of diarrhea and/or associated symptoms, or the need for treatment escalation or modification. Laboratory parameters obtained at admission included complete blood count, inflammatory markers (C-reactive protein and erythrocyte sedimentation rate), renal function, electrolytes, coagulation parameters, albumin, and lactate levels. No deaths were recorded during the 8-week follow-up period after the first CDI episode; therefore, all patients were evaluable for recurrence analysis. Both baseline (pre-CDI) and post-diagnosis variables were analyzed to assess their relative contribution to recurrence risk, as recurrence may be influenced not only by predisposing factors but also by treatment-related and disease-course characteristics.

Statistical analysis was performed using IBM SPSS Statistics, version 22 (IBM Corp., Armonk, NY, USA). Categorical variables were expressed as numbers and percentages and compared using the Chi-square or Fisher’s exact test as appropriate. Continuous variables were tested for normality and, due to non-normal distribution, were expressed as medians with interquartile ranges and compared using non-parametric tests. Receiver operating characteristic (ROC) curve analysis was used to evaluate the discriminatory ability of continuous variables associated with recurrence, with optimal cut-off values determined based on the area under the curve (AUC). Univariable and backward stepwise multivariable logistic regression analyses were performed to identify independent predictors of recurrent CDI. Odds ratios (ORs) and 95% confidence intervals (CIs) were reported. Model performance was assessed using ROC analysis of predicted probabilities. Variables with a *p*-value < 0.10 in univariable analysis were entered into the multivariable logistic regression model. Backward stepwise elimination was performed using a removal criterion of *p* > 0.05. Model calibration was assessed using the Hosmer–Lemeshow goodness-of-fit test, and internal validation was performed using bootstrap resampling techniques applied at the model level. Statistical significance was defined as *p* < 0.05.

This retrospective study was conducted using anonymized data collected from electronic medical records. According to national regulations and institutional policies, formal ethical approval was not required for this type of study. Patient confidentiality was maintained throughout the analysis.

## 3. Results

In this retrospective study, 100 patients with CDI were analyzed, of whom 18 (18%) developed recurrence. Demographic characteristics did not differ significantly between patients with and without recurrent CDI, including sex, age, body mass index, and area of residence (all *p* > 0.05).

Among healthcare-related factors, previous hospitalization within the last 12 months was significantly more frequent in patients with recurrent CDI (50.0% vs. 25.6%, *p* = 0.041). Recent hospitalization within 3 months, recent surgery, ICU admission, institutionalization, and prior nosocomial infections were not significantly associated with recurrence.

Overall, prior antibiotic exposure was not significantly associated with recurrent CDI. However, prior use of penicillins was significantly more frequent among patients with recurrence (38.9% vs. 13.4%, *p* = 0.018). No other antibiotic classes were individually associated with recurrence, although patients with recurrent CDI tended to have a higher number of antibiotic exposures prior to infection without reaching statistical significance (*p* = 0.131). Use of proton pump inhibitors, corticosteroids, chemotherapy, biological therapy, probiotics and nutritional support modalities was comparable between groups (Table 1).

Comorbidity burden, assessed by the Charlson Comorbidity Index, did not differ between groups. Individual comorbidities, including cardiovascular disease, diabetes mellitus, chronic kidney disease, pulmonary disease, malignancies, autoimmune diseases, inflammatory bowel disease, liver cirrhosis, and dementia, were not significantly associated with CDI recurrence.

Among 100 patients with CDI, 18 (18%) experienced recurrence. Clinical symptoms were generally similar between groups, with abdominal pain (38–39%) and fever (18–33%) being the most common. Severe forms and confusion were rare, and no symptom was significantly associated with recurrence (all *p* > 0.05).

Oral vancomycin was the most frequently used treatment (83–89%), followed by metronidazole. Partial treatment response was more common in recurrent cases (38.9% vs. 18.3%) but did not reach statistical significance.

Complications, including ICU transfer, sepsis and acute kidney injury, occurred more frequently in recurrent cases but were overall uncommon. Hygiene levels and prior antibiotic exposures, including fluoroquinolones, cephalosporins and multidrug-resistant pathogens, did not differ between groups. Overall, no baseline clinical feature or complication was significantly associated with recurrence; however, treatment response was significantly associated with recurrent CDI (Table 2). Table 2 summarizes the clinical presentation and outcomes of the index CDI episode stratified according to recurrence status.

Laboratory parameters at admission were similar between patients with and without recurrent CDI (Table 3). Median leukocyte counts, neutrophil percentages, hemoglobin and platelet levels did not differ significantly between groups. Inflammatory markers, including CRP and ESR, were comparable, as were renal function parameters (creatinine, urea) and electrolytes (Na, K). Coagulation parameters (INR, PT) and albumin levels were also similar. Lactate values were within normal ranges in both groups. Overall, no laboratory parameter at admission was significantly associated with CDI recurrence (all *p* > 0.05), indicating that routine blood work did not distinguish patients at risk for recurrence in this cohort.

Receiver operating characteristic (ROC) analysis identified duration of antibiotic treatment and hospitalization length as factors moderately associated with CDI recurrence (Table 4). Duration of antibiotic therapy showed the strongest discriminatory ability, with an AUC of 0.712 (95% CI, 0.585–0.839; *p* = 0.012), suggesting that longer antibiotic courses may increase the risk of recurrence. Hospitalization length was a weaker predictor, with an AUC of 0.603 (95% CI, 0.446–0.760; *p* = 0.048). These findings suggest that, among the variables analyzed, prolonged antibiotic exposure may represent a clinically relevant factor in identifying patients at higher risk for recurrent CDI (Table 4).

Backward stepwise logistic regression identified several factors associated with CDI recurrence (Table 5). In the initial model (Step 1), previous hospitalizations within the last 12 months and treatment response were significantly associated with recurrence. Patients with prior hospitalizations had a more than fourfold increased risk of recurrence (OR 4.41, 95% CI 1.30–14.98; *p* = 0.018), while poor or partial treatment response was also strongly associated with recurrence (OR 4.36, 95% CI 1.34–14.19; *p* = 0.014).

Although prior penicillin use and duration of antibiotic treatment showed positive associations with recurrence in Step 1, these did not reach statistical significance, suggesting potential confounding by healthcare exposure.

After backward elimination (Step 2), previous hospitalizations, duration of antibiotic treatment, and treatment response remained independently associated with recurrence. Each additional day of antibiotic treatment increased the odds of recurrence by 13.5% (OR 1.135, 95% CI 1.03–1.25; *p* = 0.011). Previous hospitalizations remained a strong predictor (OR 4.42, 95% CI 1.33–14.72; *p* = 0.015).

### Predictive Performance of Models

**Figure 1:**
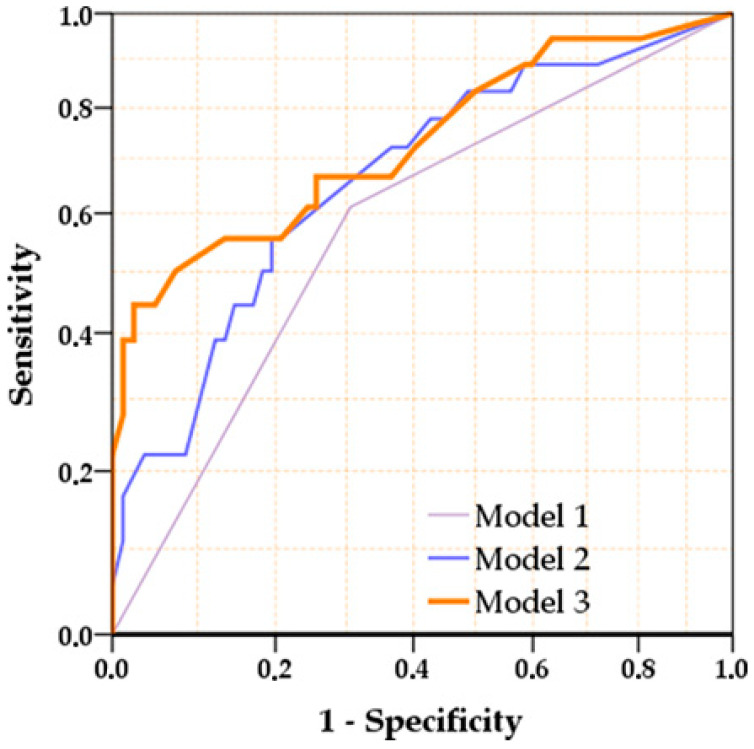
Receiver operating characteristic (ROC) curves of the stepwise predictive models for Clostridioides difficile infection recurrence. Model 1 includes treatment response alone, Model 2 incorporates treatment response and duration of antibiotic treatment and Model 3 additionally includes previous hospitalizations within the last 12 months. Progressive improvement in discriminatory performance is observed with the sequential inclusion of variables.

ROC curve analysis of the predicted probabilities derived from successive logistic regression models was performed to evaluate the incremental predictive value of the included variables (Table 6). Model 1, including treatment response alone, showed modest discrimination (AUC 0.653). The addition of duration of antibiotic treatment in Model 2 significantly improved predictive performance (AUC 0.728), indicating a meaningful contribution of antibiotic exposure to recurrence risk. Incorporation of previous hospitalizations within the last 12 months further enhanced discrimination in Model 3 (AUC 0.775), yielding the highest predictive accuracy (Figure 1).

The Hosmer–Lemeshow goodness-of-fit test confirmed adequate model calibration (χ^2^ = 7.534, df = 8, *p* = 0.480), indicating no significant difference between observed and predicted values.

The inclusion of prior penicillin use in Model 4 did not result in additional improvement in AUC, which remained unchanged (AUC 0.775). This may suggest that the univariable association of penicillin exposure with recurrence could be partly explained by variables reflecting broader healthcare exposure and antimicrobial burden (e.g., prior hospitalization and antibiotic-treatment duration); however, given the limited number of recurrence events, the multivariable model may have been underpowered to detect independent class-specific effects. A post hoc power assessment based on the observed proportions for penicillin exposure (13% vs. 39%; two-sided α = 0.05; n = 82 vs. n = 18) indicated an achieved power of approximately ~63%, suggesting moderate power for detecting this large univariable difference but limited robustness for class-specific effects after multivariable adjustment with only 18 recurrence events. Therefore, although penicillin use was associated with recurrence in univariable and regression analyses, it did not provide independent incremental predictive value beyond the variables already included in the model.

## 4. Discussions

Within our dataset, recurrence was primarily associated with markers of healthcare exposure and antibiotic burden, previous hospitalization within 12 months, longer antibiotic treatment duration and poor/partial treatment response, rather than baseline demographics, comorbidity score or admission laboratory profiles. The literature shows that recurrence occurred in 18% of cases, a proportion broadly consistent with the commonly reported recurrence range after an index episode, often between 20 and 30% [41].

Previous hospitalization within the last 12 months was significantly more frequent in patients with recurrent CDI and remained independently associated with recurrence in multivariable models. This finding is concordant with systematic evidence identifying prior hospitalization and healthcare-associated CDI as important prognostic factors for recurrence, likely reflecting repeated exposure to spores, repeated antibiotic exposure, and persistent microbiome disruption [42,43]. Conceptually, prior hospitalization may also act as a proxy for unmeasured vulnerability (frailty, repeated healthcare contacts, underlying disease instability), which is frequently captured in recurrence prediction frameworks even when individual comorbidities are not independently significant [44,45].

Although “any prior antibiotic exposure” did not significantly differ between groups, the duration of antibiotic treatment showed the strongest discrimination for recurrence and remained independently associated with recurrent CDI after adjustment. This aligns with contemporary work emphasizing that recurrence risk tracks not only antibiotic exposure per se, but also with cumulative antimicrobial pressure, which plausibly delays restoration of colonization resistance [46]. Importantly, antibiotic utilization remains the strongest modifiable determinant in CDI epidemiology and outcomes, supporting stewardship as a central lever for both primary prevention and recurrence mitigation [47].

From a mechanistic perspective, prolonged antibiotic exposure likely delays the recovery of the gut microbiome and prolongs the disruption of colonization resistance. This sustained ecological imbalance may facilitate persistence of *C. difficile* spores and increase susceptibility to relapse following apparent clinical resolution.

A higher prevalence of prior penicillin use among patients with recurrence in univariable analyses was observed, but this effect did not improve model performance once healthcare exposure and treatment duration were included. This pattern is compatible with the broader literature: comparative risk varies by antibiotic class and agent, yet class-level associations can be attenuated when cumulative exposure and healthcare-related confounding are accounted for [47]. Therefore, in practical terms, antibiotic “type” may be less informative than antibiotic “load” (duration/intensity) and the broader exposure context, particularly in small-to-moderate cohorts [43,46].

Poor/partial treatment response was independently associated with recurrence and meaningfully contributed to prediction. Clinically, this is plausible because early nonresponse may indicate persistent toxin activity, ongoing dysbiosis, inadequate host recovery or continued antibiotic exposures, all of which have been incorporated (directly or indirectly) into recurrence prediction approaches [44,45]. From a management standpoint, guidelines emphasize that treatment choices (including fidaxomicin and bezlotoxumab) should be tailored to episode type and risk of recurrence, supporting a risk-stratified approach when early clinical response is suboptimal [48].

In this cohort, admission clinical features and baseline laboratory values, including inflammatory markers, leukocyte counts, albumin, renal function, and electrolytes, were not significantly associated with recurrence. This is consistent with the notion that recurrence is often driven more by post-diagnosis exposures and microbiome trajectory than by single-time-point “snapshot” biomarkers, which have shown variable performance across settings and risk tools [49]. Moreover, meta-analytic evidence suggests that recurrence risk is influenced by factors such as age, prior hospitalization, healthcare-associated CDI and exposures occurring during/after the initial episode, highlighting the importance of longitudinal exposure measurement [42,50].

Although several meta-analyses have linked acid-suppressive therapy, particularly PPIs, to increased risk of recurrent CDI, especially when used during or after CDI, no significant association was observed [50,51]. This discrepancy may reflect limited power, differences in exposure timing (pre-CDI vs. during/after CDI), residual confounding, or local prescribing patterns [52].

Similarly, while older age and multiple comorbidities are repeatedly reported as adverse prognostic factors in broader datasets, our Charlson index and individual comorbidities were not significantly associated with recurrence, likely due to sample size constraints and collinearity with healthcare exposure markers. As no deaths occurred during the predefined 8-week follow-up period, the risk of survivorship bias related to early mortality was not present in our cohort.

These findings suggest a pragmatic risk-oriented approach in which patients with substantial healthcare exposure, prolonged antibiotic courses, and poor or partial early treatment response may represent a higher-risk subgroup. However, given the limitations of the study design and sample size, these observations should be interpreted with caution and considered hypothesis-generating rather than definitive [53,54]. This aligns with the focused update emphasizing fidaxomicin and the role of bezlotoxumab in selected patients to reduce recurrence risk [48].

In parallel, microbiota restoration is increasingly relevant in recurrent CDI management [55]. While FMT has been widely used, the therapeutic landscape now includes FDA-approved microbiota products aimed at recurrence prevention [56]. These standardized formulations may address practical limitations of conventional FMT (availability, standardization, and regulatory oversight), but continued real-world evidence is needed to define optimal sequencing and patient selection relative to antibiotics and adjunctive biologics [57,58].

Prior studies have consistently identified advanced age, antibiotic exposure, gastric acid suppression and infection with hypervirulent strains as major risk factors for recurrent CDI. In a large, hospitalized cohort, Abdelfatah et al. reported associations between recurrence and glucocorticoid use, proton pump inhibitor therapy and end-stage renal disease [59]. More recently, a nationwide study evaluating recurrence and in-hospital mortality also emphasized the role of healthcare-related factors and comorbidity burden in predicting adverse outcomes [60]. Our findings are broadly consistent with this literature in that recurrence was associated with markers of greater healthcare exposure and disease course, including prior hospitalization within 12 months, longer hospitalization duration, longer duration of antibiotic treatment, and poorer/partial clinical response during the index episode. In contrast, several commonly reported predictors (e.g., PPI use or corticosteroid therapy) were not statistically significant in our cohort, which may reflect limited statistical power given the small number of recurrent cases and the hospitalized-only study population. We therefore interpret these results as exploratory and hypothesis-generating [61].

Several limitations must be acknowledged. The retrospective design may have introduced information bias due to reliance on medical record documentation, and incomplete laboratory data cannot be excluded. Residual confounding from unmeasured variables is also possible. Selection bias may be present, as only hospitalized patients from a single center were included, potentially overrepresenting more severe cases and limiting generalizability. The relatively small number of recurrent cases, without a prior sample size calculation, may have reduced statistical power and increased the risk of model instability or overfitting. In addition, cumulative antibiotic exposure prior to CDI was not systematically quantified, and strain characteristics such as ribotype or toxin profile were not available. CDI diagnosis was based on enzyme immunoassay for toxin detection, which may have lower sensitivity compared to multistep diagnostic algorithms. Finally, although treatment response was defined based on documented clinical improvement, some degree of misclassification bias cannot be excluded. Overall, these findings should be interpreted as hypothesis-generating and require confirmation in larger, prospectively designed studies with standardized diagnostic approaches and validated predictive models.

## 5. Conclusions

In this retrospective cohort, recurrent CDI was primarily associated with healthcare exposure and treatment-related factors rather than baseline demographics, comorbidity burden, or admission laboratory parameters. Previous hospitalization, longer duration of antibiotic therapy, and poor or partial response to initial treatment remained independently associated with recurrence. These findings suggest that clinicians should prioritize careful antibiotic stewardship and close follow-up of patients with significant prior healthcare exposure or suboptimal early treatment response. However, the relatively small number of recurrent cases warrants cautious interpretation of these findings and limits the generalizability of the predictive model. Larger prospective studies are needed to validate these predictors and to refine recurrence risk stratification models.

## Figures and Tables

**Table 1:** General characteristics of the patient sample.

Variable		No CDI Recurrence (82)	Recurrence of CDI (18)	*p*-Value
**Demographic features**				
Gender	female	35 (81.4%)	8 (18.6%)	0.891
	male	47 (82.5%)	10 (17.5%)	
Age (years)		67.50 (58.00–76.00)	66.00 (56.00–75.00)	0.764
BMI (kg/m^2^)		26.55 (23.20–29.20)	27.40 (23.60–29.10)	0.476
Residency	rural	28 (84.8%)	5 (15.2%)	0.603
	urban	54 (80.6%)	13 (19.4%)	
**Healthcare exposure**				
Previous hospitalizations <12 months		21 (25.6%)	9 (50.0%)	0.041 *
Recent hospitalization <3 months		19 (23.2%)	7 (38.9%)	0.169
Recent surgery		6 (7.3%)	1 (5.6%)	0.791
Recent ICU admission		3 (3.7%)	1 (5.6%)	0.710
Institutionalized		3 (3.7%)	0 (0.0%)	0.951
Previous nosocomial infections		9 (11.0%)	1 (5.6%)	0.685
Recent antibiotics		46 (56.1%)	13 (72.2%)	0.291
**Drug use prior CDI**				
Clindamycin		4 (4.9%)	1 (5.6%)	0.905
TMP-SMX		7 (8.5%)	2 (11.1%)	0.730
Carbapenems		9 (11.0%)	4 (22.2%)	0.243
Macrolides		6 (7.3%)	3 (16.7%)	0.203
Penicillins		11 (13.4%)	7 (38.9%)	0.018 *
Fluoroquinolones		16 (19.5%)	3 (16.7%)	0.780
Number of antibiotics used		1.00 (0.00–2.00)	2.00 (0.00–2.00)	0.131
PPI		45 (54.9%)	9 (50.0%)	0.707
Corticosteroid therapy		10 (12.2%)	1 (5.6%)	0.415
Recent chemotherapy		4 (4.9%)	2 (11.1%)	0.313
Biological therapy		9 (11.0%)	2 (11.1%)	0.987
Probiotics		30 (36.6%)	7 (38.9%)	0.855
Enteral/parenteral	none	66 (80.5%)	14 (77.8%)	0.392
	enteral	11 (13.4%)	4 (22.2%)	
	parenteral	5 (6.1%)	0 (0.0%)	
**Comorbidities**				
Charlson Comorbidity Index		4.00 (3.00–5.00)	3.00 (3.00–6.00)	0.856
Diabetes mellitus		25 (30.5%)	3 (16.7%)	0.237
Arterial hypertension		38 (46.3%)	11 (61.1%)	0.256
Heart failure		10 (12.2%)	3 (16.7%)	0.609
COPD		8 (9.8%)	1 (5.6%)	0.913
Chronic kidney disease		9 (11.0%)	4 (22.2%)	0.243
Neoplasms	none	64 (78.0%)	14 (77.8%)	0.687
	remission	13 (15.9%)	2 (11.1%)	
	active	5 (6.1%)	2 (11.1%)	
Hematologic malignancies		7 (8.5%)	0 (0.0%)	0.345
Autoimmune diseases		6 (7.3%)	1 (5.6%)	0.998
Inflammatory bowel disease		8 (9.8%)	1 (5.6%)	0.913
Liver cirrhosis		2 (2.4%)	1 (5.6%)	0.452
Dementia		4 (4.9%)	1 (5.6%)	0.906

Abbreviations: CDI—Clostridioides difficile infection; BMI—body mass index; ICU—intensive care unit; TMP-SMX—trimethoprim–sulfamethoxazole; PPI—proton pump inhibitor; COPD—chronic obstructive pulmonary disease; AKI—acute kidney injury; IQR—interquartile range. “Number of antibiotics used” was defined as the total number of different antibiotic agents administered prior to the index CDI episode, while “Recent” exposures (antibiotic use, surgery, ICU admission) were defined as events occurring within 3 months prior to the index CDI episode. “Recent chemotherapy” = chemotherapy administered within 3 months prior to the index CDI episode. *: *p* < 0.05.

**Table 2:** Clinical characteristics and outcomes of the initial CDI episode.

Variable		No CDI Recurrence	Recurrence of CDI	*p*-Value
Fever		15 (18.3%)	6 (33.3%)	0.156
Abdominal pain		31 (37.8%)	7 (38.9%)	0.932
Nausea/vomiting		12 (14.6%)	3 (16.7%)	0.731
Dehydration		16 (19.5%)	1 (5.6%)	0.296
Confusion		7 (8.5%)	0 (0.0%)	0.345
Diarrhea (no. of stools/day)		6.00 (4.00–8.00)	5.00 (4.00–6.00)	0.193
Severe clinical form		5 (6.1%)	0 (0.0%)	0.582
Oral metronidazole		22 (26.8%)	6 (33.3%)	0.573
Oral vancomycin		68 (82.9%)	16 (88.9%)	0.730
Duration of hospitalization (days)		8.00 (4.00–14.00)	10.50 (6.00–15.00)	0.008 *
Duration of antibiotic treatment (days)		4.00 (0.00–10.00)	8.00 (0.00–15.00)	0.001 *
Treatment response	poor/partial	25 (30.5%)	11 (61.1%)	0.014 *
	good	57 (69.5%)	7 (38.9%)	
ICU transfer—yes		3 (3.7%)	2 (11.1%)	0.219
Sepsis—yes		2 (2.4%)	2 (11.1%)	0.089
AKI		10 (12.2%)	4 (22.2%)	0.273

Dehydration was defined based on clinical signs (e.g., dry mucous membranes, hypotension, reduced skin turgor) and/or laboratory evidence of hemoconcentration or electrolyte imbalance documented in the medical record. AKI was defined according to KDIGO criteria as an increase in serum creatinine ≥0.3 mg/dL within 48 h or ≥1.5 times baseline during hospitalization. Sepsis was defined according to Sepsis-3 criteria as life-threatening organ dysfunction associated with infection, as documented in the medical record. *: *p* < 0.05.

**Table 3:** Blood work upon admission.

Variable	No CDI Recurrence	CDI Recurrence	*p*-Value
Leukocytes (×10^3^/μL)	10.0 (7.8–11.5)	9.9 (7.5–11.3)	0.723
Neutrophils (%)	72.25 (68.2–75.2)	73.8 (69.8–77.7)	0.201
Hemoglobin (g/dL)	13.3 (11.7–14.5)	12.65 (12.2–13.7)	0.419
Platelets (×10^3^/µL)	252.5 (211–307)	258.5 (201–290)	0.925
C-reactive protein (mg/L)	26.6 (15.3–36.2)	19.4 (10.7–30.9)	0.151
ESR (mm/h)	34.2 (21.6–43.2)	33.0 (12.5–43.7)	0.641
Creatinine (mg/dL)	1.02 (0.91–1.25)	1.035 (0.80–1.69)	0.984
Urea (mg/dL)	42.8 (27.1–59.8)	48.75 (34.8–56.7)	0.545
Sodium (mmol/L)	136.3 (133.2–140.4)	137.05 (135.8–140.0)	0.122
Potassium (mmol/L)	4.1 (3.7–4.5)	3.85 (3.6–4.5)	0.366
Albumin (g/dL)	3.7 (3.4–4.0)	3.55 (3.4–3.8)	0.164
INR	1.05 (0.91–1.22)	1.15 (1.01–1.31)	0.157
Prothrombin time (s)	13.0 (11.8–14.0)	13.5 (12.3–15.2)	0.174
Lactate (mmol/L)	1.2 (0.9–1.5)	1.15 (1.0–1.4)	0.875

**Table 4:** AUC of correlated variables with CDI recurrence.

Test Variable	Area Under the Curve (AUC)	Std. Error	*p*-Value	95% CI
Duration of antibiotic treatment	0.712	0.065	0.012	0.585–0.839
Hospitalization length	0.603	0.080	0.048	0.446–0.760

**Table 5:** Backward logistic regression analysis for predictors of CDI recurrence.

Step	Variable	B Coefficient	*p*-Value	OR	95% CI for OR
1	Previous hospitalizations <12 months	1.483	0.018	4.407	1.296–14.984
1	Prior penicillin use	1.100	0.118	3.005	0.756–11.945
1	Duration of antibiotic treatment (days)	0.098	0.074	1.103	0.991–1.227
1	Treatment response (poor/partial)	1.472	0.014	4.359	1.339–14.187
2	Previous hospitalizations <12 months	1.486	0.015	4.421	1.328–14.718
2	Duration of antibiotic treatment (days)	0.126	0.011	1.135	1.029–1.251
2	Treatment response (poor/partial)	1.499	0.012	4.477	1.397–14.348

A good clinical response was defined as complete resolution of diarrhea (≤3 unformed stools per 24 h) and improvement in associated symptoms by the end of therapy, without the need for treatment modification. Poor or partial clinical response was defined as persistence or only partial improvement of diarrhea and/or associated symptoms, need for treatment escalation or modification, or lack of symptom resolution by the end of therapy.

**Table 6:** Predictive performance of multivariable models.

Model	Included Variables	AUC	*p*-Value	95% CI
Model 1	Treatment response	0.653	0.043	0.510–0.797
Model 2	Treatment response + duration of antibiotic treatment	0.728	0.003	0.595–0.861
Model 3	Treatment response + duration of antibiotic treatment + previous hospitalizations <12 months	0.775	<0.001	0.645–0.905
Model 4	Treatment response + duration of antibiotic treatment + previous hospitalizations <12 months + prior penicillin use	0.775	<0.001	0.645–0.905

## Data Availability

The datasets generated and analyzed during the current study are not publicly available due to institutional restrictions but are available from the corresponding authors upon reasonable request.
